# Emerging Roles of N6-Methyladenosine Modification in Neurodevelopment and Neurodegeneration

**DOI:** 10.3390/cells10102694

**Published:** 2021-10-09

**Authors:** Liqi Shu, Xiaoli Huang, Xuejun Cheng, Xuekun Li

**Affiliations:** 1 Department of Neurology, The Warren Alpert Medical School of Brown University, Providence, RI 02908, USA; Shuliqi@gmail.com; 2 The Children’s Hospital, School of Medicine, Zhejiang University, Hangzhou 310052, China; xiaolihuang@zju.edu.cn (X.H.); xuejun_cheng@zju.edu.cn (X.C.); 3 National Clinical Research Center for Child Health, Hangzhou 310052, China; 4 The Institute of Translational Medicine, School of Medicine, Zhejiang University, Hangzhou 310029, China; 5 Zhejiang University Cancer Center, Zhejiang University, Hangzhou 310029, China

**Keywords:** N6-methyladenosine, Mettl3, Mettl14, Fto, Ythdf1, neurodevelopment, neurodegeneration

## Abstract

N6-methyladenosine (m^6^A), the most abundant modification in messenger RNAs (mRNAs), is deposited by methyltransferases (“writers”) Mettl3 and Mettl14 and erased by demethylases (“erasers”) Fto and Alkbh5. m^6^A can be recognized by m^6^A-binding proteins (“readers”), such as Yth domain family proteins (Ythdfs) and Yth domain-containing protein 1 (Ythdc1). Previous studies have indicated that m^6^A plays an essential function in various fundamental biological processes, including neurogenesis and neuronal development. Dysregulated m^6^A modification contributes to neurological disorders, including neurodegenerative diseases. In this review, we summarize the current knowledge about the roles of m^6^A machinery, including writers, erasers, and readers, in regulating gene expression and the function of m^6^A in neurodevelopment and neurodegeneration. We also discuss the perspectives for studying m^6^A methylation.

## 1. Introduction

**Figure 1:**
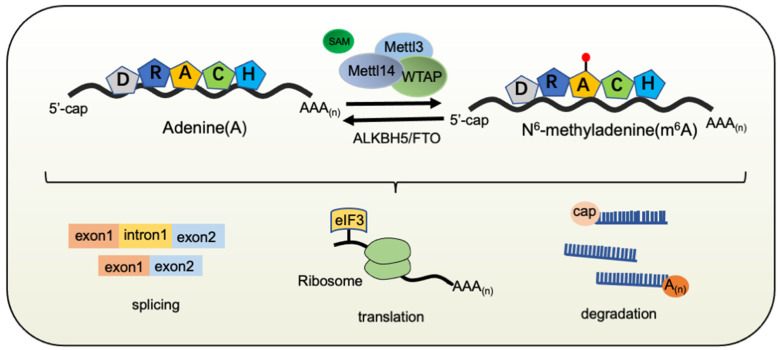
Schematic illustration of m^6^A modification. m^6^A methylation is catalyzed by the methyltransferase complex containing Mettl3, Mettl14, and an adaptor protein, such as WTAP. Fto and Alkbh5 can function as demethylases, and Yth family proteins can recognize m^6^A sites. m^6^A modification in mammals is presented on the consensus sequence DRACH (D = A/G/U, R = A/G, H = A/C/U). Reversible m^6^A modification plays important roles in regulating RNA metabolism, including RNA splicing, nuclear export, translation, and degradation in the specific context. Mettl3, methyltransferase-like 3; Mettl14, methyltransferase-like 14; WTAP, Wilms tumor 1-associating protein; Fto, fat mass and obesity-associated protein; ALKBH5, AlkB homolog 5.

Epigenetics refers to the heritable changes in gene expression and cell state caused by some specific mechanisms, aside from the occurrence of potential genetic sequences. More than 170 types of RNA modifications, including N6-methyladenosine (m^6^A), 5-methylcytidine (m^5^C), N1-methyladenosine (m^1^A), and N7-methylguanosine (m^7^G), have been identified in mammalian transcripts, and the most abundant internal RNA modification is N6-methyladenosine (m^6^A) [1,2]. m^6^A is installed by methyltransferases (writers), removed by demethylases (erasers), and recognized by m^6^A binding proteins (readers). Methyltransferase-like 3 (Mettl3) and methyltransferase-like 14 (Mettl14) form the core of the methyltransferase complex; AlkB homolog 5 protein (Alkbh5) and Fat mass and obesity-associated protein (Fto) are identified as demethylases; YTH domain family proteins (Ythdf1, Ythdf2, Ythdf3) and YTH domain-containing family protein 1 (Ythdc1) are essential reader proteins.

m^6^A modification is precisely catalyzed by a multi-subunit methyltransferase enzyme complex containing Mettl3, Mettl14, and other accessory components such as Wilms tumor 1-associated protein (Wtap), a mammalian splicing factor [3]. Mettl3 has catalytic activity, while Mettl14 acts as the RNA-binding platform and facilitates the recognition of Mettl3 [4]. Mettl3 and Mettl14 form heterodimers, which interact with Wtap. Wtap does not possess any methylation activity but interacts with Mettl3 and Mettl14 and promotes the recruitment of the Mettl3–Mettl14 complex to target transcripts [5]. The presence of m^6^A modification induces the preferential binding of certain proteins, i.e., m^6^A readers, Ythdf family proteins, and Ythdc1. In addition, m^6^A modification is reversible and can be removed by demethylases, including Fto and Alkbh5. Therefore, m^6^A machinery consists of multiple components that have diverse functions and make the field colorful (Figure 1).

m^6^A-specific methylated RNA immunoprecipitation (MeRIP) with next-generation sequencing data has revealed that m^6^A is non-randomly distributed in mRNAs but is especially enriched at the 5′ and 3′ UTRs [6,7]. m^6^A has been shown to impact RNA metabolism, including mRNA stability, translation, splicing, and localization; consequently, m^6^A regulates gene expression and involves diverse biological processes [2,8]. Present findings show that m^6^A modulates brain function [9,10] and regulates neurogenesis [11,12,13,14,15,16,17,18], brain development [7,17,18,19], axon regeneration [20], and learning and memory [13,15]. The dysregulation of m^6^A has been found in a set of neurological disorders, such as Alzheimer’s disease, Fragile X syndrome, attention-deficit/hyperactivity disorder (ADHD), and intellectual disability [19,21,22,23,24]. In this review, we summarize the recent findings regarding the function and biological consequences of m^6^A modification in the neural system, from neural development to brain function and neurological disorders.

## 2. m^6^A and Neurogenesis

### 2.1. Writers

**Figure 2:**
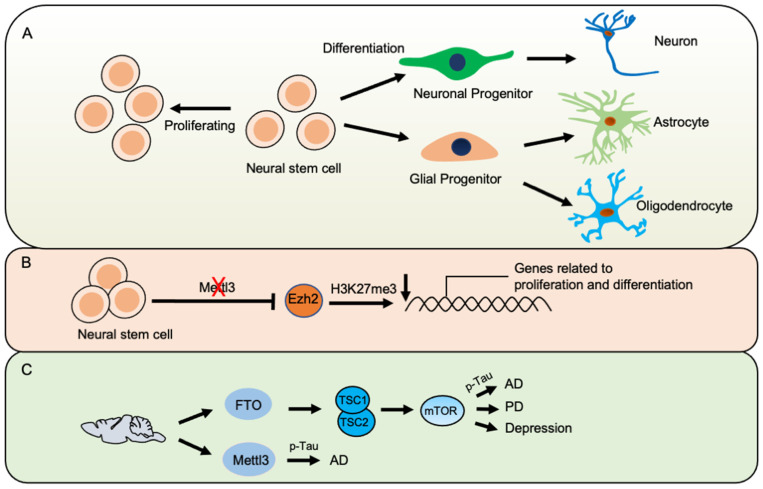
m^6^A modification in neural development and neurological disorders. (**A**). Schematic representation of neurogenesis. Neural stem cells have the capability to self-renew and differentiate into neural cells, such as neurons, astrocytes, and oligodendrocytes. (**B**). Loss of m^6^A modification affects histone modifications, including H3K27me3 and H3K27ac, which regulate the expression of genes related to the proliferation and differentiation of neural stem cells. (**C**). The modulation of m^6^A modification machinery contributes to neurodegenerative diseases, including Alzheimer’s disease and Parkinson’s disease, through the regulation of multiple pathways, such as mTOR. AD, Alzheimer’s disease; PD, Parkinson’s disease; TSC1, tuberous sclerosis 1; TSC2, tuberous sclerosis 2.

During embryonic neurogenesis, Mettl14 displays the highest expression in radial glia cells, and *Mettl14* knockout (KO) in embryonic mouse brains extends the cell cycle of radial glia cells and induces aberrant cortical neurogenesis. Similar defects were induced by Mettl3 knockdown [11]. Mettl14 also regulates the cell cycle of human cortical neuronal progenitor cells [11]. The deletion of *Mettl14* in embryonic neural stem cells (eNSCs) led to a remarkable decrease in proliferation and immature differentiation in vitro and in vivo [16]. In addition, *Mettl3* knockdown reduced the proliferation and skewed the differentiation of adult neural stem cells (aNSCs) towards neuronal lineage, while the newborn neurons displayed immature morphology [12]. Transcriptome analysis revealed that the deficiency of either *Mettl3* or *Mettl14* affected the expression of transcripts related to neurogenesis, the cell cycle, and neuronal development [11,12,16]. *Mettl3* conditional-knockout mice showed severe developmental defects of the cerebellum and cell death [17]. These results suggest an essential and conserved function of m^6^A in maintaining normal neurogenesis in the mammalian brain (Figure 2A).

m^6^A regulates gene expression not only through regulating RNA metabolism but also via modulating mRNAs encoding histone modifiers and transcription factors [25]. In mouse eNSCs, transcripts for histone acetyltransferases CBP (CREB binding protein) and p300 are m^6^A-modified [16]. In addition, transcripts for histone methyltransferase Ezh2 are also m^6^A-modified, and *Mettl3* knockdown reduces the level of Ezh2 and consequent histone H3 trimethylation at lysine 27 (H3K27me3) in aNSCs [12]. Ectopic Ezh2 could rescue *Mettl3*-knockdown-induced deficits in aNSCs [12]. These findings suggest a crosstalk between RNA modification and transcriptional regulation and reveal a new layer of the mechanism regulating neurogenesis (Figure 2C).

### 2.2. Erasers

The fat mass and obesity-associated (Fto) gene was originally referred to as an obesity-risk gene and is the first identified m^6^A demethylase [26]. The loss-of-function mutation of the *Fto* gene caused growth retardation and severe neurodevelopmental disorders, including microcephaly, functional brain defects, and delayed psychomotor activity in humans [27,28,29]. *Fto*-deficient mice showed increased postnatal mortality, significant loss of adipose tissue and body mass, and disordered energy homeostasis [27,30]. The constitutive loss of *Fto* decreased brain size and body weight, impaired the pool of adult neural stem cells (aNSCs), and impaired the learning and memory of mice [15]. Specific ablation of *Fto* in aNSCs also inhibited neurogenesis and neuronal development [13]. In addition, specific deletion of *Fto* in lipids led to decreased neurogenesis and increased apoptosis [14]. These findings indicate that Fto regulates neurogenesis through diverse pathways, including affecting brain-derived neurotrophic factor (BDNF) signaling, the expression of platelet-derived growth factor receptor (Pdgfra) and suppressor of cytokine signaling 5 (Socs5), and adenosine levels [13,14,15].

Another m^6^A demethylase, Alkbh5, is primarily localized in the nuclear speckles. Alkbh5-mediated demethylation activity affects nuclear RNA export and RNA metabolism and, consequently, regulates gene expression. The cerebellum of *Alkbh5*-deficient mice did not show detectable changes in weight and morphology, but *Alkbh5*-KO mice were more sensitive to hypoxia and showed a significantly reduced size of whole brain and cerebellum compared to control littermates [18]. In addition, the number of proliferating cells was significantly increased, but mature neurons were reduced in the cerebellum of *Alkbh5*-deficient mice [18], which suggests that *Alkbh5* deficiency affects the proliferation and differentiation of neuronal progenitor cells.

### 2.3. Readers

Ythdf1 is preferentially expressed in the hippocampus of mouse brains. Genetic deletion of *Ythdf1* impaired the learning and memory of mice, whereas it did not affect gross hippocampal and cortical histology, neurogenesis, and motor abilities [31]. Electrophysiological data showed that *Ythdf1*-deficient neurons had reduced spine density and decreased amplitude and frequency of miniature excitatory postsynaptic currents, which could be rescued by ectopic Ythdf1 [31]. This study further showed that Ythdf1 facilitates learning and memory by promoting the translation of target transcripts, including Gria1, Grin1, and Camk2a induced by neuronal stimulation.

m^6^A reader Ythdf2 is critical for embryonic development and has a lethal effect in mice [32]. *Ythdf2*-deficient mice embryos were alive at embryonic day 12.5, 14.5, and 18.5 but displayed abnormal brain development, including reduced cortical thickness and decreased proliferation of neural stem/progenitor cells (NSPCs) [32]. In addition, *Ythdf2* deficiency skewed the differentiation of NSCs towards neuronal lineage, but newborn neurons had fewer and shorter neurites [32].

Fragile X mental retardation protein FMRP can bind mRNAs, and FMRP target mRNAs are significantly enriched for m^6^A modification [22]. The loss of the FMRP coding gene *Fmr1* altered the m^6^A landscape and reduced the expression of FMRP-targeted long mRNAs in the cerebral cortex of adult mice. In addition, FMRP can interact with Ythdf2 [22]. This study provides a new layer of mechanism that specifies how FMRP regulates neuronal development and brain function.

## 3. m^6^A and Neural Development

m^6^A is abundant in the mammalian brain transcriptome, relative to other organs, and more than 25% of human transcripts are m^6^A-modified [6,7,33]. During embryonic and postnatal brain development, m^6^A displays temporal and spatial features, and specific m^6^A modification sites are present in transcripts across brain regions [6,11,21], which suggests an important role of m^6^A in neural development. Conditional deletion of *Mettl14* led to smaller sizes of newborn pups, and all died before postnatal day 25 (P25) [11]. Mettl14-cKO pups showed enlarged ventricles, delayed depletion of PAX6^+^ radial glial cells, a type of neural stem cells, and prolonged cell-cycle progression [11]. Similar phenotypes were also observed in the brains of embryonic mice with *Mettl3* knockdown [11]. m^6^A sequencing showed that transcripts with m^6^A modification were related to the cell cycle and neuronal differentiation [11]. In addition, during the postnatal cerebellum development, the global level of m^6^A decreases from P7 to P60, and m^6^A is developmentally/temporally modulated [18]. Specific m^6^A peaks at P7 were close to stop codon regions, whereas P60-specific m^6^A peaks were near start codons [18]. *Mettl3* deficiency induces embryonic lethal effects, and the acute knockdown and specific ablation of *Mettl3* both induced remarkable cortical and cerebellar defects, including a reduced number of Purkinje cells and the increased apoptosis of cerebellar granule cells [17,18].

*Fto*-deficient mice showed a decreased body weight compared to control mice, and the sizes of whole and distinct brain regions were also decreased remarkably [15]. In contrast to control mice, which exhibited locomotor activity induced by cocaine, *Fto*-deficient mice significantly lost their response to cocaine [34]. Mechanistically, Fto can also demethylase mRNAs involved in dopamine signaling, including Ped1b, Girk2, and Syn1; consequently, Fto can alter dopamine midbrain circuitry [34]. *Alkbh5*-knockout mice also showed drastically smaller cerebella and reduced mature neurons [18]. Collectively, these findings highlight the critical function of m^6^A in neural development.

## 4. m^6^A in Axonal and Synaptic Development

Acute knockdown of *Mettl3* led to remarkable decreases of newborn neurons upon the differentiation of aNSCs, which displayed an immature morphology, with a reduced number of intersections and decreased total dendritic length [12]. In addition, *Mettl3* knockdown also inhibited the morphological development of cultured hippocampal neurons [12]. Fto was enriched in the dendrites and synapses of neurons and can be locally translated into axons [35]. Treatment with a Fto activity inhibitor promoted m^6^A signals but inhibited axon elongation by regulating the axonal translation of Gap-43 [36]. In addition, transcripts for Roundabout (Robo) family member Robo3.1, an axon guidance receptor, were m^6^A-modified, and m^6^A reader Ythdf1 regulated axon guidance via the promotion of the translation of Robo3.1 [37]. Beyond affecting axon growth, m^6^A also regulates axon regeneration. Peripheral nerve injury induces a dynamic m^6^A landscape and enhances the expression of mRNAs modified by m^6^A, including Sox11, Atf3, and Gadd45a [20]. *Mettl14* ablation in mature neurons promoted the translation in the adult dorsal root ganglion (DRG) and reduced the length of the longest neuronal process [20]. Similar effects were also observed in adult DRGs of *Ythdf1*-KO mice.

In addition, m^6^A modification that was identified in the synaptic transcriptome and in transcripts with m^6^A peaks in the stop codon but not in the start codon are associated with neurological dysfunction, including intellectual disability, microcephaly, and seizures [38]. m^6^A level was negatively correlated with transcript abundance in synaptosomal RNAs, suggesting the local degradation of m^6^A mRNA [38]. Interestingly, m^6^A peaks in the stop codon did not show a strong effect on the synaptic location of transcripts [38]. Furthermore, in contrast to hypomethylated transcripts, hypermethylated transcripts were highly related to synaptic development and neurological disorders, including intellectual disability, autism, and schizophrenia. [38].

## 5. m^6^A and Gliogenesis

Astrocytes and oligodendrocytes are two major macroglia cells in the brain that account for at least 50% of brain cells and are involved in diverse biological processes and brain function. In addition, to induce abnormal neurogenesis, acute knockdown of *Mettl3* induces precocious astrocytes upon the differentiation of NSCs [12]. Constitutive deletion of *Mettl14* can significantly reduce astrogenesis in embryonic mice brains [11]. Furthermore, *Ythdf2*-deficient NSCs only generate neuronal cells but not glial cells upon the differentiation [32]. Genetic ablation of *Ythdf2* also increased the sensitivity of newborn neurons to reactive oxygen species stress [32]. Mechanistically, the expression of some transcripts related to neural development and differentiation, axon guidance, and synapse development (i.e., Nrp2, Nrxn3, Flrt2, Ptprd, Ddr2) was remarkably upregulated in *Ythdf2*-deficient NSCs [32]. One identified mechanism is that *Ythdf2* deficiency represses m^6^A-modified mRNA clearance [32]. These findings indicate that m^6^A writers and reader(s) are essential for the proper temporal progression of neurogenesis and gliogenesis.

In addition to its important roles in astrocytes, differential m^6^A peaks were detected in transcripts during the differentiation of oligodendrocyte precursor cells (OPCs) to mature oligodendrocytes. Specific inactivation of *Mettl14* in oligodendrocytes reduces the number of mature oligodendrocytes and, consequently, leads to hypomyelination [39]. Furthermore, *Mettl14* deficiency inhibits oligodendrocyte differentiation, including morphological development, but does not affect OPCs. One potential mechanism is that the loss of *Mettl14* induces the abnormal splicing of myriad RNA transcripts, including neurofascin 155 [39]. Proline-rich coiled-coil 2A (Prrc2a) is a novel m^6^A reader and is highly expressed in OPCs. *Prrc2a* deficiency reduces the proliferation of OPCs and decreases the expression of oligodendroglial lineage-related transcripts via the direct modulation of the half-life of Olig2 mRNA [40]. Consequently, *Prrc2a*-deficient mice exhibited hypomyelination and impaired locomotive and cognitive abilities [40].

## 6. m^6^A and Brain Function

Specific deletion of *Mettl3* in CaMKIIα-expressing neurons impairs long-term potentiation, which enhances long-term memory consolidation via the modulation of the translation of immediate-early genes, such as Arc, Egr1, and c-Fos [41]. Genetic ablation of *Mettl14* in dopamine D1 receptor (D1R)-expressing striatonigral neurons or dopamine D2 receptor (D2R)-expressing striatopallidal neurons also decreased the expression of neuron- and synapse-specific proteins, decreased the number of striatal cells double-labeled for mature neuronal marker NeuN and Mettl14, and increased neuronal excitability [42]. Behavioral tests show that *Mettl14* deficiency in these two types of neurons impairs sensorimotor learning and reversal learning [42].

The constitutive or NSC-specific deletion of *Fto* not only causes aberrant neurogenesis, it also impairs the learning and memory abilities of mice [13,15]. In addition, fear condition training induced dynamic m^6^A modification, and the majority peaks were present in mRNAs. *Fto*-specific knockdown in the mouse medial prefrontal cortex (mPFC) enhanced the cued fear memory [43]. *Ythdf1*-KO mice exhibit deficits in spatial learning and memory and contextual learning [31]. *Ythdf1* deficiency also impaired basal synaptic transmission and long-term potentiation of mice, which can be rescued by ectopic Ythdf1 [31]. Ythdf1 modulates learning and memory formation mainly by promoting the translation of neuronal-stimulation-related transcripts. Heat shock stress can specifically increase m^6^A modification in 5′UTR and can alter the cellular localization and expression of Ythdf2, but not Fto, Mettl3, Mettl14, and Wtap [44]. The level of m^6^A modification in 5′UTR was correlated with the expression of a set of transcripts, especially the Hsp70 gene *Hspa1a* [44].

## 7. m^6^A and Neurological Disorders

Consistent with important functions in neural development [18,32], neurogenesis [11,12,15,16], learning and memory [12,13,15,42] and stress response [44,45], the present evidence also indicates that m^6^A modification is involved in several neurological disorders, including Alzheimer’s disease (AD), and Parkinson’s disease (PD), schizophrenia, and attention-deficit/hyperactivity disorder (ADHD) via the regulation of gene expression and RNA metabolism [10,11,46,47,48,49,50]. Next, we discuss the function of m^6^A modification in neurodegenerative diseases, including Alzheimer’s disease and Parkinson’s disease.

A temporal feature of m^6^A modification has been revealed during postnatal brain development and aging [6,12]. In the brain of amyloid precursor protein (APP)/presenilin-1 (PS1) (APP/PS1) transgenic AD mouse models, m^6^A levels increased in the cortex and hippocampus, and the expressions of Mettl3 and Fto increased and decreased, respectively, compared with control mice [48]. Very recently, Shafik et al. found that m^6^A peaks decreased during the maturation stage of postnatal brain development (postnatal 2 weeks to 6 weeks), whereas these peaks increased during the process of aging (26 weeks and 52 weeks) [21]. In addition, this study also showed increased Fto expression and decreased Mettl3 expression. The differentially methylated transcripts were enriched in the signaling pathways related to Alzheimer’s disease, and differential m^6^A methylation is associated with decreased protein expression in an AD mouse model, which was further validated in a Drosophila transgenic AD model [21]. In agreement with this study, the Fto protein level increased in the brain tissues of transgenic AD mice, and Fto depletion did not affect the level of amyloid β 42 (Aβ42) but significantly increased the level of phosphorylated Tau in the neurons from an AD mice model [51]. They further found that Fto regulates Tau phosphorylation by activating mTOR signaling. Yoon et al. performed MeRIP, followed by next-generation sequencing with forebrain organoids, and the ontology analysis of human-specific m^6^A-targeted transcripts showed an enrichment in neurodegenerative disorders, including Alzheimer’s disease [11]. Taken together, these findings suggest that m^6^A modification could play a pivotal function in the progression of AD (Figure 2C).

Acute knockdown of *Mettl14* in substantia nigra reduced m^6^A levels and impaired motor function and locomotor activity [52]. Nuclear receptor-related protein 1 (Nurr1), pituitary homeobox 3 (Pitx3) and engrailed1 (En1) are related to tyrosine hydroxylase expression and dopaminergic function, and their expression was remarkably reduced by *Mettl14* depletion [52]. The specific knockout of *Fto* in dopaminergic neurons impairs the dopamine neuron-dependent behavioral response by regulating dopamine transmission, which implies the important role of Fto-mediated m^6^A demethylation in regulating dopaminergic midbrain circuitry [34]. In a Parkinson’s disease (PD) rat model, the overall level of m^6^A in the striatum decreased, and the Fto level significantly increased [53]. Either ectopic Fto or treatment with m^6^A inhibitors reduces m^6^A levels and induces oxidative stress and apoptosis of dopamine neurons, partially by promoting the expression of N-methyl-D-aspartate (NMDA) receptor 1 [53]. Consistently, *Fto* knockdown increases m^6^A levels and reduces apoptosis in vitro [53]. In addition, a large cohort study with 1647 Han Chinese individuals with Parkinson’s disease (PD) has identified 214 rare variants in 10 genes with m^6^A modification; however, no significant association was observed between these variants and the risk for PD according to their analysis [54]. Therefore, the roles of m^6^A modification still need more comprehensive investigation (Figure 2C).

## 8. Conclusions and Perspectives

As the most abundant modification in mRNAs, previous studies have revealed the dynamic features of m^6^A modification and have uncovered its important function in a variety of biological processes and diseases. It seems that the more we explore m6A modification, the more complicated it becomes. First, m^6^A modification is reversible and includes multiple key “players”: writers, erasers, and readers. The interaction between these key players and other epigenetic modifications, such as histone modifiers, makes the field more complicated. Second, the complexity of m^6^A modification also lies in the fact that it is hard to define a promoting or repressing function of m^6^A modification in a set of diseases. The deficiency of m^6^A writers and erasers could show similar effects on the diseases but could not exhibit contrary effects as routinely thought. Third, m^6^A modification can regulate a defined biological process, i.e., the maintenance, renewal, and differentiation of neural stem cells by modulating diverse gene expression and signaling pathways. In addition, multiple players of m^6^A modification exhibit effects on the same biological process, such as neurogenesis. It is hard to distinguish whether the effect is independent of each other, and it remains unclear whether they crosstalk. Therefore, how m^6^A writers, erasers, and readers cooperate to regulate adult neurogenesis still needs more investigation.

Although dramatic progress has been made in understanding the function of m^6^A modification, future studies should devote more effort to uncovering the multi-faceted nature of the associated mechanisms. The interaction between m^6^A modification and histone modifiers suggests a colorful landscape wherein m^6^A modification interacts with other epigenetic machinery, i.e., DNA modifications and non-coding RNAs. In addition, considering a substantial enrichment of m^6^A in the 5′ and 3′ UTRs of transcripts, do multiple writers, erasers, and readers have binding specificity for distinct regions? Finally, establishing a more precise spatiotemporal landscape of m^6^A in the pathological context could be of clinical significance. With the technical advances of sequencing, we anticipate the identification of key m^6^A site(s) that can contribute to the diagnosis and treatment of specific diseases.
